# Wernicke encephalopathy complicating catatonic schizophrenia

**DOI:** 10.1192/j.eurpsy.2021.1399

**Published:** 2021-08-13

**Authors:** C. Rihab, S. Ellouze, N. Messedi, M. Turki, F. Ben Ali, W. Abid, M. Halouani, J. Aloulou

**Affiliations:** 1 Psychiatry (b), Psychiatry (B), Hedi Chaker University hospital, sfax, Tunisia; 2 Psychiatry (b), Hedi Chaker University hospital, sfax, Tunisia

**Keywords:** Wernicke’s encephalopathy, catatonic schizophrenia, Korsakoff syndrome

## Abstract

**Introduction:**

Wernicke’s encephalopathy is a potentially fatal neurological emergency caused by thiamine deficiency. Although it is often associated with chronic alcoholism, it can also occur in all situations that lead to a thiamine deficiency such as undernutrition and exclusive artificial feeding.

**Objectives:**

In this work, we propose to study the clinical and treatment concerns of Wernicke’s encephalopathy complicating catatonic schizophrenia.

**Methods:**

We retrospectively report the case of a patient who developed a Wernicke’s encephalopathy in the aftermath of catatonic schizophrenia.

**Results:**

Mr H.L, a 47-year-old-male has been followed in psychiatric hospital since the age of 27 for catatonic schizophrenia. He has been hospitalized in July 2020 because of oral intake refusal, social isolation and lack of self-care with a poor compliance to treatment. Examination of the patient revealed catalepsy, mutism and negativism. He was treated with antipsychotics drugs, benzodiazepines and parenteral nutrition. About six weeks after his hospitalization, the patient developed horizontal nystagmus and ataxic gait. Magnetic resonance imaging was consistent with Wernicke encephalopathy. Vitamin B1 dosage was 32nmol/l. Parenteral thiamine replacement therapy was initiated with clinical improvement

**Conclusions:**

Catatonic schizophrenia can be associated with severe malnutrition and thus with thiamine deficiency and Wernicke’s encephalopathy. An early intervention by supplying prophylactic thiamine given parenterally in high-risk patients is crucial to avoid Korsakoff syndrome, as well as cardiovascular and neuropsychiatric complications associated with thiamine deficiency.

